# Children’s and Mothers’ Perspectives of Problematic Eating Behaviours in Young Children and Adolescents: An Exploratory Study

**DOI:** 10.3390/ijerph16152692

**Published:** 2019-07-28

**Authors:** Kamila Czepczor-Bernat, Anna Brytek-Matera

**Affiliations:** Katowice Faculty of Psychology, SWPS University of Social Sciences and Humanities, 40-326 Katowice, Poland

**Keywords:** problematic eating behaviours, BMI, young children, adolescents, mothers

## Abstract

The aim of this study was to (a) compare children’s perspectives of problematic eating behaviours with those of mothers and (b) check if there are differences in the level of these problematic eating behaviours between girls and boys in different age groups (young children: 8–11 years old vs. adolescents: 12–16 years old). The study involved 203 children (50.74% girls) and 203 mothers. The average age of children was 11.06 years (SD = 2.31), and the average BMI was 18.27 kg/m^2^ (SD = 2.29). Two questionnaires were used to assess children’s perspectives of problematic eating behaviours: The Three-Factor Eating Questionnaire (TFEQ-R13) and the Dutch Eating Behaviour Questionnaire for Children (DEBQ-C). One questionnaire was used to evaluate mothers’ perspectives: The Child Eating Behaviour Questionnaire (CEBQ). The main results in the study indicate the following: (a) the children’s perspective based on the DEBQ-C is the most effective at predicting their BMI (this model of problematic eating behaviours explains 29% of the variance in the child’s BMI); and (b) for almost all problematic eating behaviours, older girls have the highest levels. From the current study, it can be concluded that the type of questionnaire (TFEQ-R13 vs. DEBQ-C vs. CEBQ) and the perspective (child vs. mother) differentiate the results obtained regarding the assessment of children’s problematic eating behaviours and their relation to BMI.

## 1. Introduction

A significant body of evidence indicates that problematic eating behaviours are an important factor related to the body mass index (BMI) in children and adolescents [1,2,3]. The term “problematic eating behaviours” includes many types of eating behaviours that adversely affect somatic and mental health (including leading to an increase in BMI) [4,5,6]. Among problematic eating behaviours, the literature distinguishes among the following [3,4,7,8,9]: (a) the tendency to eat when experiencing negative emotions and stress; (b) eating without feeling in control and without taking into account the level of hunger and satiety; (c) high reactivity to external triggers under the influence of which the amount of consumed food increases; and (d) the tendency to restrict food intake.

At this point, it is worth noting that the results of previous studies on the relationship between problematic eating behaviours and BMI are not consistent. On the one hand, in studies with an increase in BMI, the level of restrictive eating increases and the level of emotional and external eating decreases [3,10]. On the other hand, research by Baños et al. [1] shows that the higher BMI is the greater emotional and restrained eating becomes. Moreover, similar inconsistencies occur in studies in which girls and boys as well as older and younger children are compared in terms of the intensity of problematic eating behaviours. In the studies of Nagl et al. [11], women have a higher level of restrictive and emotional eating compared to men, and younger children eat in a more restrictive, uncontrolled and emotional way than the older group does [12]. On the basis of other studies [1,13], it has been concluded that girls and boys as well as older and younger children do not differ in the context of emotional overeating, food responsiveness and satiety responsiveness. The aforementioned inconsistencies require further research that takes into account the fact that their source may also be (a) using various questionnaires to measure problematic eating behaviours or (b) using different perspectives in the assessment of children’s problematic eating behaviours (perspective of a child vs. perspective of a parent).

There are many questionnaires used in the assessment of children’s eating behaviour. However, very often discussed in the literature are, among others, the Three-Factor Eating Questionnaire (TFEQ) [2,12,14,15], the Dutch Eating Behaviour Questionnaire for Children (DEBQ-C) [1,3,10,11,16,17] and the Child Eating Behaviour Questionnaire (CEBQ) [13,18,19].

In summary, in many studies, the relationship between problematic eating behaviours and BMI has been analysed [1,3,10]. However, these studies have not verified the following: (1) which of the currently used questionnaires assessing abnormal eating styles (TFEQ or DEBQ-C or CEBQ) are the most effective at predicting a child’s BMI; and (2) whether the perspective of a child or mother (in the assessment of problematic eating behaviours) is the most effective at predicting a child’s BMI. Therefore, the first aim is to compare the child’s perspective of problematic eating behaviours (two questionnaires: TFEQ vs. DEBQ-C) with the mother’s perspective (one questionnaire: CEBQ). Moreover, because of the inconsistency among the results from other studies (e.g., [1,11,12,13]), the second aim of the study is to determine whether there are differences in the level of problematic eating behaviours between girls and boys in different age groups (young children: 8–11 years old vs. adolescents: 12–16 years old) using various questionnaires (TFEQ, DEBQ-C, CEBQ).

## 2. Materials and Methods

### 2.1. Participants

At the first stage of the cross-sectional descriptive study (T1), 203 children (50.74% girls) and 203 mothers participated in the research. By means of a probabilistic sampling method, the research included children who met the following criteria: (a) agreeing to take part in research by the informed consent form, which was signed by children and parents; (b) children between 8 and 16 years of age; and (c) not meeting criteria of feeding and eating disorders. After applying the above criteria, 203 dyads (from 220) participated in the study (6 children suffered from feeding and eating disorders; 11 dyads returned questionnaires with numerous data gaps).

The average age of children was 11.06 years (SD = 2.31), and the average BMI was 18.27 kg/m^2^ (SD = 2.29). The demographic characteristics of both age groups, based on Erikson’s stages of psychosocial development for young children vs. adolescents, are shown in Table 1.

Finally, it should be emphasized that to determine the adequate number of participants needed to address the research aims, the sample size was calculated (effect size = 0.15, power = 0.95, *α* = 0.05) [21]. A power analysis (using the G*Power tool) indicated that the study should involve a minimum of 119 participants [22].

### 2.2. Measures

The following questionnaires were used:

(1) The Three-Factor Eating Questionnaire (TFEQ-R13) [2] is a self-applied Polish version of a 13-item questionnaire measuring three types of problematic eating behaviours: (a) emotional eating: “When I feel anxious, I find myself eating”; (b) uncontrolled eating: “When I see a real delicacy, I often get so hungry that I have to eat right away”; and (c) restrictive eating: “I deliberately take small helpings as the means of controlling my weight”. For items 1–12, the children respond on a 4-point Likert scale (from 1—“definitely false” to 4—“definitely true”). For item 13 (8-point Likert scale), responses range between 1—“eating whatever you want, whenever you want it” to 8—“constantly limiting food intake and never ‘giving in’” (1–2 are recoded into 1; 3–4 into 2; 5–6 into 3; and 7–8 into 4). The higher the score is, the higher the intensity of problematic eating behaviours. Cronbach’s alpha reliability coefficients were as follows: (a) emotional eating: 0.70; (b) uncontrolled eating: 0.70; and (c) restrictive eating: 0.86.
(2) The Dutch Eating Behaviour Questionnaire for Children (DEBQ-C) [17] is a 20-item questionnaire and measures the three subscales of problematic eating behaviours: (a) emotional eating: “If you feel depressed, do you get a desire for food?”; (b) external eating: “Do you feel like eating whenever you see or smell good food?”; and (c) restrained eating: “Do you intentionally eat food that helps you lose weight?”. Responses range (5-point Likert scale) from 1 (“never”) to 5 (“very often”). The lower the scores are, the lower the problematic eating behaviours are. The present study focused on all of the abovementioned subscales, and their Cronbach’s alpha values were as follows: (a) emotional eating: 0.84; (b) external eating: 0.71; and (c) restrained eating: 0.88.
(3) The Child Eating Behaviour Questionnaire (CEBQ) [19] is a 20-item questionnaire that measures parents’ assessment of the eating behaviours of their children. The scale is composed of eight subscales: (a) food responsiveness: “If allowed to, my child would eat too much”; (b) emotional over-eating: “My child eats more when annoyed”; (c) enjoyment of food: “My child enjoys eating”; (d) desire to drink: “My child is always asking for a drink”; (e) satiety responsiveness: “My child cannot eat a meal if s/he has had a snack just before”; (f) slowness in eating: “My child eats slowly”; (g) emotional under-eating: “My child eats less when upset”; and (h) food fussiness: “My child is difficult to please with meals”. Response options range (5-point Likert scale) between 1 “never” and 5 “always”. The present study focused on the three types of eating behaviours (food responsiveness, emotional over-eating and satiety responsiveness) that are semantically similar to the subscales described in the TFEQ-R13 and the DEBQ-C. Higher scores for food responsiveness and emotional over-eating indicate more problematic eating behaviours. The scale of satiety responsiveness is interpreted inversely: High scores are more adaptive eating styles. Cronbach’s alpha reliability coefficients were as follows: (a) food responsiveness: 0.83; (b) emotional over-eating: 0.70; and (c) satiety responsiveness: 0.84.
(4) The sociodemographic survey involves questions about gender, age, height, weight, feeding and eating disorders. The children’s anthropometric data were collected in a survey completed by the mothers.
(5) The Eating Disorders in Youth-Questionnaire (EDY-Q) is a self-report 14-item measure assessing feeding and eating disorders [23]. Items were created based on the Diagnostic and Statistical Manual of Mental Disorders, Fifth Edition (DSM-5) [24]. This tool was used to exclude children who had symptoms of feeding and eating disorders.

### 2.3. Procedure

Prior to the beginning of the study, the consent of the local ethics committee was obtained (no. 50/03/2017). Next, all questionnaires’ authors authorised the use and translation of their measurements. Therefore, a standard forward–backward translation procedure was used to produce the Polish version of the DEBQ-C, the CEBQ and the EDY-Q (Dzielska et al. [2] created a Polish version of the TFEQ). Before the application of the final version of all questionnaires, competent judges (e.g., developmental psychologists, teachers of children aged 8–16 years old, school counsellors) assessed and confirmed them. Then, parents and children were given a set of the abovementioned questionnaires to complete. Before completing all questionnaires, parents were asked to measure the height and weight of their children and complete the sociodemographic data. Next, children completed the TFEQ-R13, the DEBQ-C and the EDY-Q, and mothers completed the CEBQ. Rewards and incentives were not offered to children and mothers.

This is an exploratory study (paper-and-pencil assessment) because its objectives are related to a problem that before has not been explored and/or the results of other studies are inconsistent. Thus, there is no theoretical basis for establishing detailed hypotheses on the perspective of the individual (child or mother) who is the most effective at predicting BMI and the group (younger girls vs. younger boys vs. older adolescent girls vs. older adolescent boys) with the highest level of problematic eating behaviours. This is the first stage of longitudinal research that is funded by the Polish National Science Centre (no 2017/25/N/HS6/00004). This is an important stage of the project because the results and conclusions obtained at this point will be used in further stages of the project.

### 2.4. Data Analysis

The Statistical Package for Social Sciences version 22.0 was used. Before statistical analyses (described below) were performed, reliability (Cronbach’s alpha) and the structure of the questionnaires (principal components analysis) were verified. They were satisfactory and similar to the findings of the original report [2,17,19,23]. Therefore, multivariate regression analysis was used to verify the relationship between problematic eating behaviours and BMI from a child–mother dyadic perspective. For the children’s perspective assessment, the TFEQ-R13 subscales and the DEBQ-C subscale were used separately. Regarding the perspective of mothers, it was decided to limit the comparison of mothers’ models to three subscales of the CEBQ (given that the value of “Y” depends on the number of predictors, i.e., the formula of the regression equation is Ŷ = *B*_0_ + *B*_1_ • *X*_1_ + *B*_2_ • X_2_ + *B*_3_ • *X*_3_) [25]. Because of the exploratory study, according to Tabachnick and Fidell [26], the enter method for multivariate regression analysis was utilized. Analysis of variance (ANOVA) was used to compare girls and boys in the two age groups (young children vs. adolescents) in terms of the TFEQ-R13, the DEBQ-C and the CEBQ subscales. A conservative post hoc Bonferroni correction was introduced.

## 3. Results

### 3.1. Children’s and Mothers’ Perspectives of the Relationships Between Problematic Eating Behaviours and BMI: Multivariate Regression Analysis

In relation to the first aim of this study, three regression analyses were performed (the first two models regarding the children’s perspectives and the third model regarding the mothers’ perspectives). The first model is associated with the assessment of the BMI predictors, which are subscales of the TFEQ-13 (emotional eating, uncontrolled eating and restrictive eating) (*F*(3, 199) = 16.76, *p* < 0.001). The second model involved BMI predictors such as emotional eating, external eating and restrained eating (DEBQ-C) (*F*(3, 199) = 27.82, *p* < 0.001). The last model, which is related to the CBEQ, included three predictors: Food responsiveness, emotional over-eating and satiety responsiveness (*F*(3, 199) = 14.14, *p* < 0.001). Table 2 shows the results of these analyses.

### 3.2. Comparison Between Girls and Boys in Different Age Groups: ANOVA

**Figure 1:**
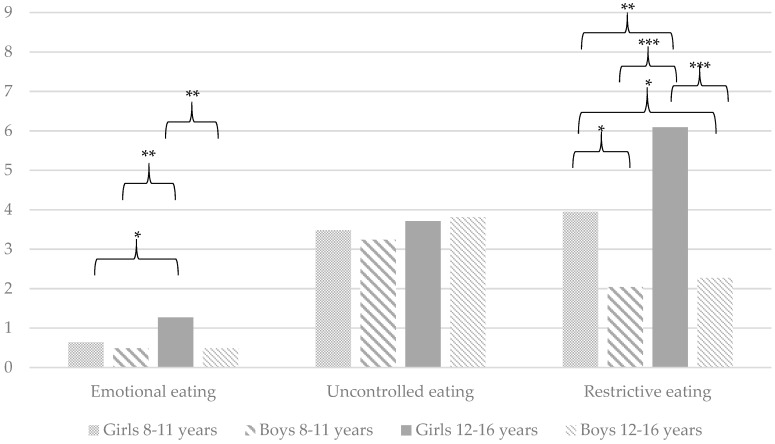
A child’s perspective: Mean problematic eating behaviours measured by the Three-Factor Eating Questionnaire (TFEQ-R13). * *p* < 0.05, ** *p* < 0.01, *** *p* < 0.001. Only statistically significant differences are marked on the graph.

**Figure 2:**
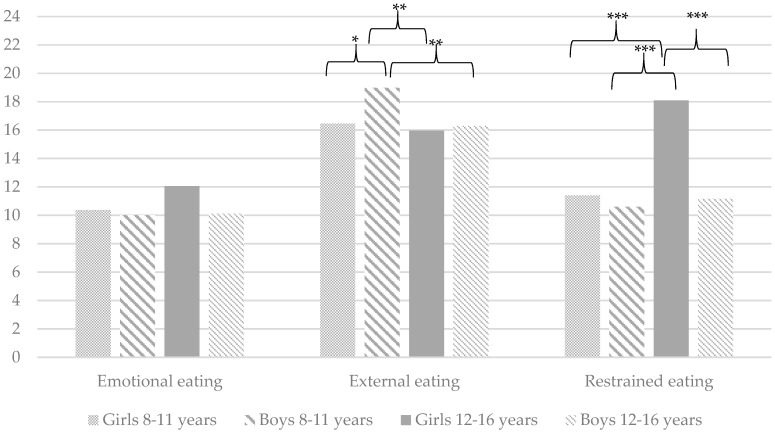
A child’s perspective: Mean problematic eating behaviours measured by the Dutch Eating Behaviour Questionnaire for Children (DEBQ-C). * *p* < 0.05, ** *p* < 0.01, *** *p* < 0.001. Only statistically significant differences are marked on the graph.

**Figure 3:**
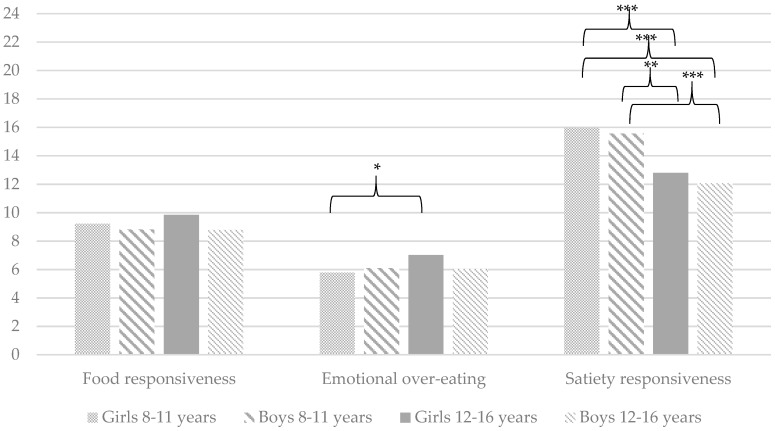
A mother’s perspective: Mean problematic eating behaviours measured by the Child Eating Behaviour Questionnaire (CEBQ). * *p* < 0.05, ** *p* < 0.01, *** *p* < 0.001. Only statistically significant differences are marked on the graph.

As a second aim of this study, it was determined whether there are differences in the level of problematic eating behaviours between girls and boys in different age groups (young children: 8–11 years old vs. adolescents: 12–16 years old). Analysis of variance shows significant differences between the abovementioned groups on the following variables: (a) TFEQ-R13: Emotional eating (*F*(3, 199) = 5.46, *p* < 0.01) and restrictive eating (*F*(3, 199) = 14.74, *p* < 0.001); (b) DBEQ-C: External eating (*F*(3, 199) = 5.21, *p* < 0.01) and restrained eating (*F*(3, 199) = 18.38, *p* < 0.001); and (c) CEBQ: Emotional over-eating (*F*(3, 199) = 3.29, *p* < 0.05) and satiety responsiveness (*F*(3, 199) = 16.05, *p* < 0.001). In terms of other variables, the groups do not differ significantly on the following: (a) TFEQ-R13: Uncontrolled eating (*F*(3, 199) = 0.63, *p* > 0.05); (b) DEBQ-C: Emotional eating (*F*(3, 199) = 2.52, *p* > 0.05); and (c) CEBQ: Food responsiveness (*F*(3, 199) = 0.85, *p* > 0.05). Figure 1, Figure 2 and Figure 3 provide detailed comparisons between girls and boys in different age groups.

## 4. Discussion

With regard to the first objective, the present study provides support for the perspective of the child based on the DEBQ-C as being most effective at predicting children’s BMI because this perspective explains 29% of the variance in children’s BMI (while the TFEQ-R13 explained 19% of the variance and the CEBQ explained 16% of the variance). Moreover, the best predictor of BMI is restricted eating from the DEBQ-C, followed by restrictive eating from the TFEQ-R13. This may indicate the particular importance of this style of eating for BMI in children and adolescents. From the children’s perspective, other statistically significant predictors (except for food restrictions) are uncontrolled eating (TFEQ-R13) and external eating (DEBQ-C). From the mothers’ perspective, the important predictors are emotional eating and correct reaction on satiety. Interestingly, emotional eating is not significant from the children’s perspective. In summary, the results related to the first objective of the study indicate that the next stage of this project and future research should take into account the perspectives of both children and mothers in the assessment of problematic eating behaviours (with consideration given to the following: (a) BMI will most accurately predict the DEBQ-C questionnaire, and (b) the parents’ perspective may be helpful in the assessment of the relationship between emotional eating and BMI). Moreover, for all significant predictors (except for the “satiety responsiveness” subscale, which is interpreted inversely), the higher their levels are, the higher the BMI is. The current findings are partly consistent with those of previous research showing that the more restrictive eating habits are, the higher the BMI is [1,4,10]. However, our research did not confirm that the relationship between BMI and external and emotional food is negative [1,10].

In relation to the second aim of this study, it is observed that girls and boys in two age groups (young children: 8–11 years old vs. adolescents: 12–16 years old) differ in the context of problematic eating behaviours. Both the TFEQ-R13 and DEBQ-C show that participants differ in the level of restrictive eating. The most important findings in this context are summarised as follows: (a) the highest level is observed in older girls, followed by younger girls; and (b) younger and older boys do not differ from each other. However, more significant differences among all analysed groups can be found using the TFEQ-R13. This finding shows, therefore, that restrictive eating is more intense among girls than among boys, and its intensity increases significantly with age. Interestingly, our findings confirm earlier results in relation to gender-related comparisons [11] but contradict the results regarding the comparisons of age groups [12].

Only the TFEQ-R13 and CEBQ show significant differences between groups in the level of emotional (over-)eating. Again, both questionnaires coherently indicate that the highest level of this type of problematic eating behaviour is characteristic of older girls. However, only on the TFEQ-R13 is the result obtained by them significantly higher than those obtained by all others (the CEBQ indicates a significant difference between younger and older girls). Our results are confirmed by the study of Nagl et al. [11], which indicated that women have a higher level of emotional eating. However, our results negate other findings [12] that indicate that younger children show higher levels of emotional eating.

One of the possible explanations of the results regarding restrictive and emotional eating is the development of emotional awareness and self-control in children and adolescents [27,28]. These two characteristics develop with age, which can be connected, on the one hand, with the fact that emotions become triggers to eat. On the other hand, the frequency of introducing eating restrictions also increases (to achieve weight reduction). Such a pattern of problematic eating behaviours may lead to the development of eating disorders, which, as other studies show, are the most disseminated among adolescent girls [29,30].

Finally, it should be mentioned that the highest level of external eating (DEBQ-C) is observed among younger boys and the highest level of a correct reaction to the feeling of fullness is observed among younger girls (the results of both groups differ significantly from those of the other three groups). These results confirm the above assumption (regarding eating disorders) that for girls over time, weight control through restrictive eating begins to have greater significance than physiological signals related to hunger and satiety and external triggers. The lack of significant between-group differences for emotional eating (DEBQ-C), uncontrolled eating (TFEQ-R13) and food responsiveness (CEBQ) is consistent with the findings of some previous research [1,13].

These studies are valuable because there are few studies in Poland regarding the assessment of the eating behaviours of children and adolescents and BMI from a child–mother dyadic perspective (e.g., [31]). However, a few limitations of the current study should be mentioned, which should be addressed in subsequent studies. First, a more objective and detailed assessment of body weight should be used (e.g., body composition by use of bioelectrical impedance analysis) among children and mothers. Second, the relationship between problematic eating behaviours and BMI should be verified in longitudinal studies. Third, based on other studies [1,32], in the future (next to the division into sex and age), body weight should be categorized as either normal or excessive to compare the analyses carried out in these two groups. Fourth, apart from the perspective of mothers, it is necessary to consider the perspective of fathers. Fifth, in future research it may be interesting to speculate on the possibility to collect information on food choices of children. Sixth, it would be interesting to examine how the mothers’ BMI influences the BMI of children and their eating behaviours. All these indications will be included by the authors of this article in the further stages of the project.

In conclusion, this study showed certain findings that are consistent with those of many studies. These findings include a positive relationship between restrictive eating and BMI and a higher level of this type of problematic eating behaviour among girls than among boys. This finding may also indicate that restrictive eating is a characteristic of children and youth regardless of the cultural context. Other discoveries are divergent, which suggests that intercultural research in this context may prove extremely valuable in the investigation of the mechanism underlying these results.

It is well-known that eating behaviours established in childhood persist, with implications such as fussiness and poor dietary variety or high responsiveness to food cues and increased obesity risk [33]. It is worth pointing out that nutrition policies and obesity prevention in Poland should include the need to educate children and adolescents in the context of eating behaviours, especially in young female adolescents and those with restrictive eating.

## 5. Conclusions

The following main conclusions can be drawn from the current study: (a) for the first aim, the perspective of the child based on the DEBQ-C is the most effective at predicting children’s BMI; and (b) for the second aim, older girls have the highest level of almost all problematic eating behaviours. The results for both aims confirmed that the type of questionnaire (TFEQ-R13 vs. DEBQ-C vs. CEBQ) and the perspective (child vs. mother) differentiate the results obtained regarding the assessment of children’s problematic eating behaviours and their relation to BMI.

## Figures and Tables

**Table 1:** Descriptive statistics for the two age groups.

Variables	Young Children (8–11 years)	Adolescents (12–18 years)
	*N* (%)	
	104	99
Sex ^1^		
Female	59 (56.73)	44 (44.44)
Male	45 (43.27)	55 (55.56)
	*M* (*SD*)	
Age	9.14 (1.03)	13.07 (1.37)
Height	139.45 (6.78)	158.67 (8.14)
Weight	34.25 (5.63)	48.13 (8.38)
BMI ^2^	17.54 (2.07)	19.03 (2.27)
	*N* (%)	
BMI		
Underweight	2	3
Normal weight	80	88
Overweight	17	7
Obesity	5	1

^1^ The number of girls and boys in both age groups did not differ significantly, *χ*^2^(3, *N* = 203) = 3.25; *p* > 0.05. ^2^ The children’s body mass index (BMI) was calculated based on the Polish growth chart (percentile analysis) [20]. Projects “OLA” and “OLAF” were used as the basis for calculating the BMI of children [20].

**Table 2:** A child–mother dyadic perspective: An analysis of the predictors of BMI.

Predictors	*β*	*R* ^2^ *-Change*	
TFEQ-R13		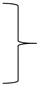	0.19
Emotional eating	−0.112		
Uncontrolled eating	0.158 *		
Restrictive eating	0.432 ***		
DEBQ-C		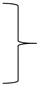	0.29
Emotional eating	−0.116		
External eating	0.154 *		
Restrained eating	0.570 ***		
CEBQ		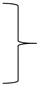	0.16
Food responsiveness	0.009		
Emotional over-eating	0.349 **		
Satiety responsiveness	−0.197 **		

* *p* < 0.05, ** *p* < 0.01, *** *p* < 0.001.
